# Protective Effects of *Moringa oleifera* on HBV Genotypes C and H Transiently Transfected Huh7 Cells

**DOI:** 10.1155/2017/6063850

**Published:** 2017-10-26

**Authors:** Sina Feustel, Fabiola Ayón-Pérez, Ana Sandoval-Rodriguez, Roberto Rodríguez-Echevarría, Homero Contreras-Salinas, Juan Armendáriz-Borunda, L. V. Sánchez-Orozco

**Affiliations:** ^1^Instituto de Biología Molecular en Medicina y Terapia Génica, Departamento de Biología Molecular y Genómica, Centro Universitario de Ciencias de la Salud, Universidad de Guadalajara, Guadalajara, JAL, Mexico; ^2^Tecnológico de Monterrey, Guadalajara, JAL, Mexico

## Abstract

Chronic hepatitis B infection treatment implicates a long-lasting treatment. *M. oleifera* extracts contain compounds with antiviral, antioxidant, and antifibrotic properties. In this study, the effect of *M. oleifera* was evaluated in Huh7 cells expressing either HBV genotypes C or H for the antiviral, antifibrotic, anti-inflammatory, and antioxidative responses. Huh7 cells were treated with an aqueous extract of *M. oleifera* (leaves) at doses of 0, 30, 45, or 60 *μ*g/mL. The replicative virus and *TGF-β1*, *CTGF*, *CAT*, *IFN-β1*, and pgRNA expressions were measured by real time. HBsAg and IL-6 titers were determined by ELISA. *CTGF*, *TGF-β1*, *IFN-β1*, and pgRNA expressions decreased with *M. oleifera* treatment irrespective of the HBV genotype. HBsAg secretion in the supernatant of transfected Huh7 cells with both HBV genotypes was decreased regardless of the dose of *M. oleifera*. Similar effect was observed in proinflammatory cytokine IL-6, which had a tendency to decrease at 24 hours of treatment. Transfection with both HBV genotypes strongly decreased *CAT* expression, which is retrieved with *M. oleifera* treatment. *M. oleifera* treatment reduced fibrosis markers, IL-6, and HBsAg secretion in HBV genotypes C and H. However, at the level of replication, only HBV-DNA genotype C was slightly reduced with this treatment.

## 1. Introduction

Hepatitis B virus (HBV) belongs to the Hepadnaviridae family. It is a noncytopathic and hepatotropic virus and causes inflammation, cirrhosis, and, eventually, hepatocellular carcinoma (HCC). There are around 2 billion people infected with the virus worldwide, and more than 240 million people are chronic carriers of HBV [1]. There are ten different recognized genotypes of HBV. In Mexico, the most prevalent genotype is H followed by G and A [2, 3]. When HBV infects a host cell, the partial double-stranded DNA is repaired generating the covalently closed circular DNA (cccDNA), and then, this DNA is transcribed into different mRNAs; one is the pregenomic RNA (pgRNA). This RNA represents a replication intermediate and is reverse transcribed into viral DNA by viral polymerase. Together with translated surface antigens (HBsAg), the virus is able to leave the host cell for further infection [4, 5].

Nowadays, there are five antiviral drugs (lamivudine, adefovir, entecavir, telbivudine, and tenofovir) and IFN (conventional or pegylated) approved by the United States Food and Drug Administration for HBV treatment. According to international guidelines, treatment with antiviral drugs implicates a long-lasting administration. On one side, in the United States, treatment is for the entire life, while in Europe, treatment is prescribed until complete virus elimination and antigen disappearance. During long-term treatment, there is a risk of developing resistant HBV mutants. Favorable IFN treatment is limited to less than 30% of the patients. Treatment success is defined with normalization of functional hepatic tests, seroconversion, decrease of viral load, and histological improvement. The loss of HBsAg is the best predictor of sustained remission after treatment but is not achieved very frequently with the actual HBV treatment [6–11].

The *Moringa oleifera* (*M. oleifera*) tree belongs to the family of Moringaceae [12]; its origin is in the north of India, and the cultivation is possible in tropical and subtropical countries [13]. The different parts of the tree are used especially in traditional medicine. Extracts of leaves of *M. oleifera* have shown the highest antioxidant activity [14]. Additionally to antioxidative properties, *M. oleifera* leaves are rich in vitamins, carotenoids, polyphenols, phenolic acids, flavonoids, alkaloids, glucosinolates, isothiocyanates, tannins, saponins/oxalates, and phytates [15]. There is only one report about antiviral effect of *M. oleifera* in cccDNA of HBV with no conclusive evidence [16]. In that paper, buffered and alcoholic extracts of *M. oleifera* (leaves) were evaluated as a preventive therapy. Since, extracts were added previously to transfection at HepG2 cells and only cccDNA was measured. In our study, *M. oleifera* was added after transfection of Huh7 cells, and antiviral, antifibrotic, and antioxidative responses in Huh7 cells expressing genotypes C or H of HBV were evaluated.

## 2. Materials and Methods

### 2.1. *M. oleifera*

The *M. oleifera* leaves were obtained from the south coast of Jalisco, Mexico and were validated by the Instituto Nacional de Investigaciones Forestales, Agrícolas y Pecuarias (INIFAP). The leaves were pulverized and dissolved in sterile water at a final concentration of 10 mg/mL. This aqueous extract was left to sit for 65 h at 4°C in the dark and was centrifuged 2 times at 13,000 ×g for 10 min, and then the supernatant was filter-sterilized (0.2 *μ*m). The extracts were stored at −80°C.

### 2.2. Cell Culture

Huh7 cells were maintained at 37°C and 5% CO_2_ in Dulbecco's Modified Eagle Medium (Gibco™) supplemented with 10% fetal bovine serum (Gibco) and 1% penicillin/streptomycin (Gibco). Medium was changed every third day, and when cells reached a confluence of 80 to 90%, they were passaged to a new culture flask.

### 2.3. Viability

5 × 10^4^ Huh7 cells were seeded in 6-well plates. After 24 h of culture, the first dose of aqueous *M. oleifera* extract (0, 30, 45, 60, 120, 250, and 500 *μ*g/mL) was added and repeated two times every 24 h until cell viability was determined at 72 h with a Guava ViaCount (Guava Technologies) flow cytometer. The cell concentration of stained cells was between 1 × 10^4^ and 5 × 10^5^ cells/mL in accordance with the recommendations of the manufacturer.

### 2.4. Plasmid Construction

The complete genome of HBV genotype H was amplified from DNA obtained from serum of an infected patient. The full genome of HBV was cloned in pGEM-T EASY (Promega). The clones were analyzed by restriction enzymes. Clones with the HBV were selected to subclone into pHY-106 vector (kindly donated by William Delaney). This vector contains the minimum HBV sequence necessary for viral transcription and replication after insertion of a full length HBV genome [17]. Then, selected clones (pHY-H) were analyzed by restriction enzymes (*EcoRI*/*XhoI*) (ChemiDoc™ XRS+, Bio-Rad), and DNA sequencing was performed to verify the integrity of the insert. The plasmids were analyzed by CLC Sequence Viewer and genotypified by NCBI genotyping. Plasmids with the complete genome of genotype C cloned into pHY-106 vector were a kind gift from Dr. Jake Liang.

### 2.5. Transfection

2 × 10^5^ Huh7 cells were seeded in 6-well plates. When the cells reached a confluence of 80 to 90%, cells were transfected transiently with 0.7 *μ*g of plasmid pHY-C, pHY-H, or pHY-106 (control) and lipofectamine 2000 (Invitrogen) according to the manufacturer's instruction. Transfection efficiency and green fluorescent protein (GFP) were detected by fluorescent microscopy. All transfections were performed in triplicate.

### 2.6. *M. oleifera* Treatment

After 6 h of transfection, cells were washed with PBS, then DMEM plus either 0, 30, 45, or 60 *μ*g/mL of *M. oleifera* was added. This treatment was repeated further at 24 and 48 h, and the supernatant was stored at −80°C. Cells were harvested after 72 h.

### 2.7. Quantitative Real-Time Reverse Transcriptase PCR

RNA extraction was performed with TRIzol reactive according to the manufacturer's instruction. Briefly, 500 ng of total RNA was treated with DNase I (Thermo Fisher Scientific) and reverse transcribed using M-MLV (Invitrogen) according to the manufacturer's instruction, respectively. The resulting cDNA was used for real-time PCR using LightCycler® 96 (Roche), LightCycler TaqMan® Master (Roche), and appropriate primers for pgRNA (S: 5′-GTT CAT GTC CYA CTG TTC AAG CC, AS: 5′-TAG AGG GCT GAA GCG GTG TC). *CAT* (S: 5′-CTG ACA CTC ACC GCC ATC GCC, AS: 5′-GCT GTG CTC CAG GGC AGA AGG), *CTGF* (S: 5′-AAT GCT GCG AGG AGT GGG, AS: 5′-TGG CTC TAA TCA TAG TTG GGT CT), and *TGF-beta1* (S: 5′-CAC TGC TCC TGT GAC AGC AG, AS: 5′-GGT GGC CAT GAG AAG CAG GA) and *IFNbeta1* (Applied Biosystems: Hs01075529_m1). The relative expression was normalized by *GAPDH* (S: 5′-CAT GAG AAG TAT GAC AAC AGC CT, AS: 5′-AGT CCT TCC ACG ATA CCA AAG T) expression.

### 2.8. HBV-DNA Quantification

A mixture from the triplicates from the cell culture supernatant of transfected Huh7 cells was prepared with proportional volumes. DNA was extracted from 200 *μ*L of the mixture using QIA amp minElute Virus Spin Kit (Qiagen). 2.5 *μ*L was used as a template for real-time PCR using 480 SBYR Green I master (Roche Molecular Systems, Inc.), according to the manufacturer's instruction. Two standard curves were generated: one for HBV-DNA quantitation and the other to quantify the plasmid to be subtracted to avoid interference with the quantity of the viral DNA. Appropriated primers were PHY-106, S: 5′ GTAAACTGCCCACTTGGCAG, R: 5′ AGCGATGACTAATACGTAGATG. HBV, S: 5′ TTCGCAGTCCCCAAYCTC (310-327), and R: 5′ CAMACGGGCAACATACCTTG (474-455).

### 2.9. Secretion of HBsAg and IL-6

HBsAg secretion was detected with Monolisa™ HBsAg ULTRA (Bio-Rad) in 100 *μ*L of the cell culture supernatant of transfected Huh7 cells according to the manufacturer's instruction. The OD was read at 450/700 nm. IL-6 was determined by competitive ELISA (R&D Systems) according to the manufacturer's instructions. Levels of IL-6 and HBsAg were determined in an ELISA reader (*μ*Quant Bio-Tek, Instruments, Inc.).

### 2.10. Statistical Analysis

To compare the data between two independent groups, the *t*-test was used. For no parametric data, Kruskal-Wallis and Mann–Whitney *U* tests were applied. Values with ^∗^*p* < 0.05 were considered statistically significant. For statistical analysis, SPSS version 20 and Microsoft Office Excel 2010 were used.

## 3. Results

### 3.1. Viability

**Figure 1:**
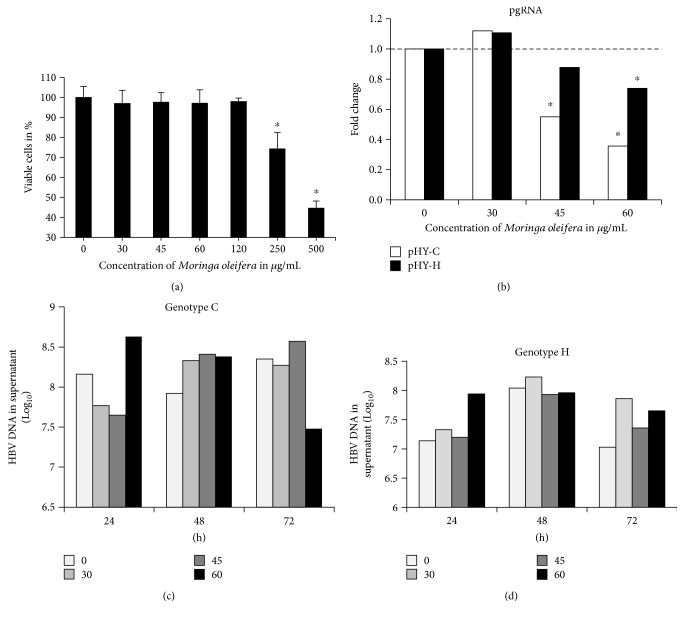
(a) Effect of aqueous leaf extract of *Moringa oleifera* on the viability of Huh7 cells. ^∗^*p* < 0.05. (b) pgRNA expression of Huh7 cells transfected with genotype C (white bars) or H (black bars) of HBV and treated with *Moringa oleifera*. Fold change (2^−ΔΔCt^) was calculated with reference data without treatment. ^∗^*p* < 0.05 based on ΔCt values. (c and d) HBV-DNA was quantified from supernatants recovered at 24, 48, and 72 hours after treatment by real-time PCR (copy number expressed in Log_10_); *x*-axis specifies the *Moringa oleifera* dose at *μ*g/ml.

The aqueous *M. oleifera* extract decreased the viable cell number (*p* < 0.05) at concentrations of 250 and 500 *μ*g/mL (74.3 and 44.7%, resp.) (Figure 1(a)). In general, the lower the concentration of *M. oleifera*, the lower was the number of death cells. Concentrations of 30, 45, 60, and 120 *μ*g/mL of *M. oleifera* decreased the number of viable cells in an average of 2.8%. Thus, for further experiments, the three lowest concentrations of *M. oleifera* (30, 45, and 60 *μ*g/mL) were used to test our proof of concept.

### 3.2. Pregenomic RNA Expression

There was a slight tendency of pgRNA expression reduction in cell cultures of transfected cells with HBV genotypes C and H and the treatment of 30 *μ*g/mL of *M oleifera* (Figure 1(b)). The doses of 45 and 60 *μ*g/mL of *M. oleifera* reduced (*p* < 0.05) the pgRNA expression with HBV genotype C-transfected cells (0.55 and 0.36, resp.). Cells transfected with HBV genotype H showed a decrease (0.74, *p* < 0.05) at the highest concentration of *M. oleifera* studied (60 *μ*g/mL).

### 3.3. HBV-DNA Quantitation

Treatment with *M. oleifera* reduced HBV-DNA from genotype C, 1.09 Log_10_ at 60 *μ*g/mL at 72 h of treatment (Figure 1(c)). Viral DNA from genotype H was not reduced by the treatment at any dose and time (Figure 1(d)).

### 3.4. HBsAg Secretion

**Figure 2:**
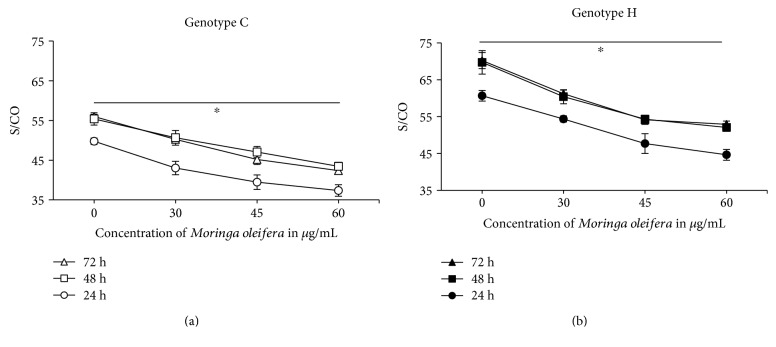
HBsAg secretion after *Moringa oleifera* treatment. Huh7 cells transfected with genotype C (a) and genotype H (b) of HBV and treated with 0, 30, 45, and 60 *μ*g/mL of *Moringa oleifera*. ^∗^*p* < 0.05.

The HBsAg secretion in the supernatant of transfected Huh7 cells with genotypes C and H of HBV was decreased (*p* < 0.05) independently of the dose of *M. oleifera* treatment (Figures 2(a) and 2(b)). At 24 h, the HBsAg secretion was lower than at 48 and 72 h, though *M. oleifera* treatment displayed the same effect. The ratio of immunoassay signal strength of the sample to cut-off (S/CO) at 24 h without treatment was 49.8 with genotype C of HBV. *M. oleifera* treatment of 30, 45, and 60 *μ*g/mL reduced the S/CO to 43.0, 39.5, and 37.3, respectively. This data represents a decrease up to 25.1% of HBsAg secretion with the highest dose of *M. oleifera* at 24 h. At 48 and 72 h, the reduction of secretion was similar (21.5 and 24.3%, resp.). Compared with genotype H of HBV, HBsAg secretion was higher but treatment with *M. oleifera* also reduced the S/CO value. At 24 h, the S/CO without treatment was 60.6 and decreased with *M. oleifera* treatment of 30, 45, and 60 *μ*g/mL (54.4, 47.7, and 44.6, resp.). Independently of the time point of measurement, *M. oleifera* treatment reduced the HBsAg secretion between 24.6 and 26.4% (72 and 24 h, resp.).

### 3.5. CTGF and TGF-*β*1 Gene Expression

**Figure 3:**
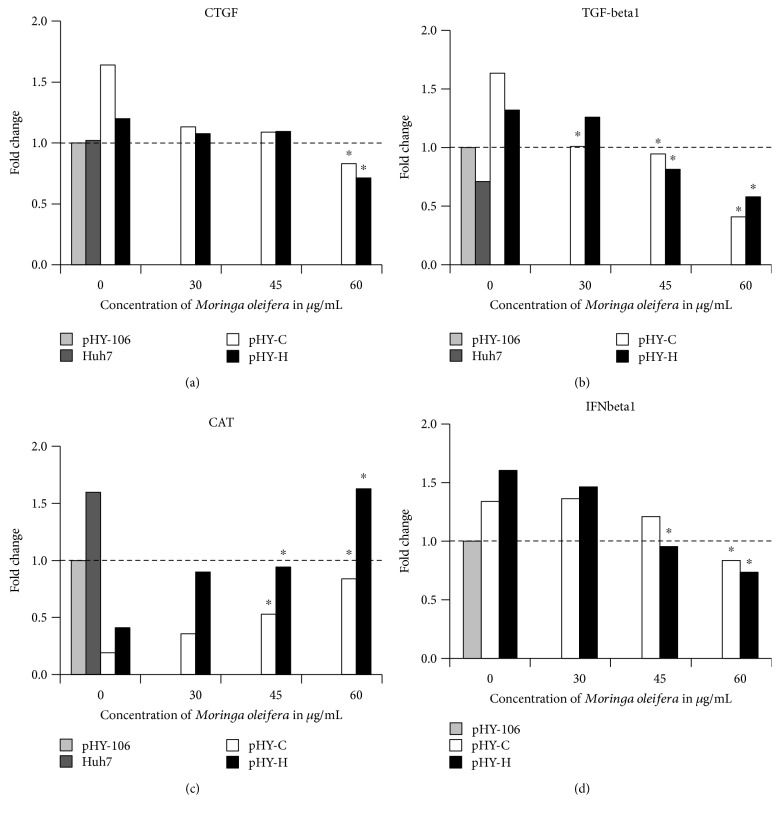
(a) *CTGF*, (b) *TGF-beta1*, (c) *CAT*, and (d) *IFNbeta1* gene expressions of Huh7 cells transfected with genotype C (white bars) or H (black bars) of HBV and treated with *Moringa oleifera*. Fold change (2^−ΔΔCt^) was calculated with reference data without treatment and plasmid pHY-106 (without HBV genome). ^∗^*p* < 0.05 based on ΔCt values.

The gene expression of *CTGF* and *TGF-β1* decreased with *M. oleifera* treatment independently of the genotype of HBV (Figures 3(a) and 3(b)). There was no difference in *CTGF* expression in Huh7 cells compared with Huh7 cells transfected with the plasmid without HBV insert (pHY-106; Figure 3(a)). Transfection of the cells with genotypes C and H of HBV increased the expression (1.64 and 1.2 times, resp.). *M. oleifera* treatment with 60 *μ*g/mL significantly reduced *CTGF* expression (genotype C up to 0.83 times and genotype H up to 0.71 times). *TGF-β1* expression was induced by the transfection of the plasmid without HBV insert (pHY-106) compared with Huh7 cells (0.71 < 1; Figure 3(b)). The transfection with HBV genotypes C and H enlarged this induction of expression (1.63 and 1.32 times, resp.). *M. oleifera* treatment of 30, 45, and 60 *μ*g/mL significantly reduced *TGF-β1* expression with genotype C of HBV (1.01, 0.95, and 0.41, resp.). With genoytpe H of HBV, the decrease (*p* < 0.05) of *TGF-β1* expression started with 45 *μ*g/mL of *M. oleifera* treatment (0.81 and 0.58 times with 60 *μ*g/mL dose).

### 3.6. CAT Gene Expression

The gene expression of *CAT* was decreased in Huh7 cells transfected with the plasmid without HBV insert (pHY-106) compared with Huh7 cells (1 > 1.59; Figure 3(c)). Transfection with genotypes C and H of HBV strongly decreased *CAT* expression (0.19 and 0.41 times). Interestingly, treatment with *M. oleifera* restored this reduction (*p* < 0.05) starting with 45 *μ*g/mL dose with both genotypes of HBV. The increase of *CAT* expression was more marked with genotype H than with genotype C of HBV (45 *μ*g/mL dose 0.94 > 0.53 and 60 *μ*g/mL dose 1.63 > 0.84).

### 3.7. IFNbeta1 Gene Expression

The gene expression for *IFNβ1* was increased by transfection of Huh7 cells with HBV genotypes C and H compared with the plasmid without insert (pHY-106; 1 < 1.34 < 1.60; Figure 3(d)). *M. oleifera* treatment reduced the *IFNβ1* expression (*p* < 0.05) with 45 and 60 *μ*g/mL doses with HBV genotype H (0.95 and 0.74 times, resp.). With genoytpe C of HBV, the highest dose of *M. oleifera* treatment decreased (*p* < 0.05) the *IFNβ1* expression (0.83 times).

### 3.8. IL-6 Quantitation in the Supernatant of Huh7-Transfected Cells

**Figure 4:**
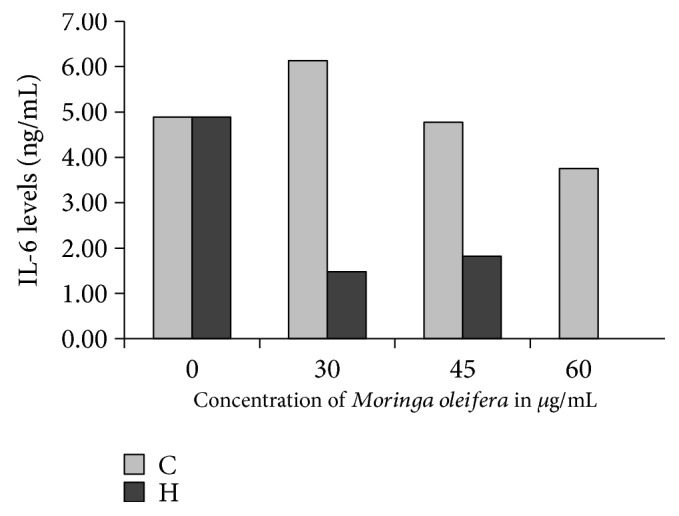
IL-6 levels after *Moringa oleifera* treatment determined in the supernatant of Huh7 cells transfected with genotypes C and H. A mixture from the triplicates from the cell culture supernatant of transfected Huh7 cells was prepared with proportional volumes. ELISA was performed with a capture-specific mouse anti-human IL-6 antibody (100 *μ*L at 2 *μ*g/mL). After incubation and washing steps, detection was performed with Biotinylated Goat anti-human IL-6 detection antibody (100 *μ*L at 50 ng/mL) using streptavidin-HRP.

IL-6 levels determined by ELISA in the supernatant of transfected Huh7 cells were detected only at 24 hours (genotype C from 3.7 to 6.14 ng and genotype H from 0 to 4.89 ng). The quantitation of IL-6 at 48 and 72 hours after *M. oleifera* did not show positive values. Interestingly, after *M. oleifera* treatment, IL-6 showed a tendency to decrease in a dose-dependent manner in the supernatant of Huh7-transfected cells with genotypes C and H (Figure 4).

## 4. Discussion

The viability of Huh7 cells was not affected with the aqueous extract of the *M. oleifera* leaves at the concentrations of 30, 45, 60, and 120 *μ*g/mL after 72 h (Figure 1). There is no report about the cytotoxic effect of *M. oleifera* in Huh7 cells. Waiyaput et al. [16] studied HepG2 and COS-7 cell viability after 5 days of treatment with an ethanolic leaves extract of *M. oleifera* (50, 150, and 300 *μ*g/mL). The viable cell number of HepG2 cells decreased about 15.5, 27.9, and 65%, respectively. Compared with COS-7 cells, the cell viability was reduced significantly in comparison with HepG2 cells with the treatment of 150 *μ*g/mL (63.5 > 27.9%). In a different study, Sangkitikomol et al. [18] reported the cell viability of HepG2 cells treated with an ethanolic extract of *M. oleifera* leaves at 29 h. *M. oleifera* concentrations of 100, 200, and 400 *μ*g/mL had no cytotoxic effect on the cells, and higher concentrations, such as 600, 800, and 1000 *μ*g/mL, induced only a slight tendency in cell death (5–10% of viable cell reduction). Regarding this issue, scientific literature shows us a report about cell viability of HeLa, HepG2, MCF-7, CACO-2, and L929 cells at 24 h with treatment with an essential oil of *M. oleifera* seeds [19]. In all cell lines, viability decreased with the increase of *M. oleifera* concentration. Our results shown here are in accordance with the published data. Cell viability is affected by the *M. oleifera* in a dose-dependent manner. The different extracts showed at various times of measurement an increase of cytotoxicity in distinct cell lines. The extract preparation is not standardized between the studies. In dependence of the preparation of the extract and the buffer used, there is a divergence of components in the extracts. This variation of the components can cause distinct cytotoxic effects in the same cell line.

The pgRNA expression decreased with an increase of *M. oleifera* treatment in accordance with the HBsAg secretion, which was reduced independently of *M. oleifera* concentration and HBV genotype (Figure 2). There are no reports about the effect of *M. oleifera* on pgRNA expression and/or HBsAg secretion. There is only one study published about HBV and *M. oleifera* [16]. They measured the cccDNA expression in HepG2 cells treated with a buffer or alcoholic extract of *M. oleifera* leaves and further transfected with HBV. The alcoholic extract of *M. oleifera* (30 *μ*g/mL) had no effect on the cccDNA expression, meanwhile 0.3 *μ*g of total protein of a buffer extract of *M. oleifera* decreased significantly the cccDNA expression (0.2 times). The methodology of the experiment varies with our study. In the present study, we transfected first Huh7 cells with two different genotypes of HBV and then treated with *M. oleifera*. Thus, our proof of concept was rationalized in a different manner, since we studied *M. oleifera* effect as a therapeutic agent instead of a preventive action as Waiyaput et al. [16] reported. Additionally, our data shows that pgRNA did not differentiate the replicative virus from the transfected plasmid. However, it is very important to state that the drop in pgRNA levels were statistically evidenced by repeating the experiment three times in triplicate cultures as well as by real-time PCR. Nevertheless, this pgRNA level reduction was only supported by a slightly reduction in HBV-DNA levels in the supernatant of Huh7 cells transfected with genotype C at 60 *μ*g/mL after 72 hours of treatment (Figure 1(c)). Probably, the reduction observed in the pgRNA of genotype H was not enough to reduce the rate of replication of the virus, but the antifibrotic and antioxidant effects deduced by the changes in *CTGF*, *TGFβ*, and *CAT* gene expressions with *M. oleifera* treatment support the fact that Moringa might be used as a valuable nutritional supplement for patients with chronic hepatitis B. There are additional studies, both *in vivo* and *in vitro*, reporting effects of *M. oleifera* in different viruses, like herpes simplex virus type 1 [20], Epstein-Barr virus [21], Newcastle disease virus [22], and infectious bursal disease virus [23]. Nevertheless, the complete elucidation of the molecular mechanisms driving *M. oleifera* action remains to be defined.

*CTGF* expression was not modified by transfection with the plasmid without HBV insert (pHY-106) in contrast to an increase of expression when transfected with HBV (Figure 3(a)), though expression of *TGF-β1* was enhanced in cells transfected with the HBV-less plasmid (pHY-106; Figure 3(b)). This effect was amplified when cells were transfected with HBV genotypes C and H. *M. oleifera* decreased the expression of *CTGF* and *TGF-β1* in Huh7 cells transfected with genotypes C and H of HBV (Figures 3(a) and 3(b)).

HBV infection causes a profibrogenic environment with increase of CTGF and TGF-*β*1 protein expressions in human hepatic stellate cells (HSCs) cocultivated with HBx [24]. Pan et al. reported an increase of TGF-*β*1 protein expression in HepG2 cells expressing HBxAg [25]. *CTGF* and *TGF-β1* expressions activate HSCs and triggers a profibrogenic response, increase of extracellular matrix, and expression of collagen 1 alpha [26, 27]. In this study, we detected antifibrogenic potential of *M. oleifera*. There is no data about the possible mechanism of regulation of *CTGF* and *TGF-β1* expressions by *M. oleifera*. However, it is known that quercetin is one of the components of *M. oleifera* [28, 29], and our group [30] reported the potential quercetin effect to regulate the balance of pro- and antifibrogenic stimuli, rendering reduction of activated HSCs and ameliorating fibrosis in rats.

Quercetin in *M. oleifera* leaves moderates inflammation of high-fat-fed mice [28]. Park and Chang [31] showed a decrease of mRNA and protein expression of fibronectin, type I collagen, and plasminogen activator inhibitor I in a rat kidney fibrosis model. On the other hand, hepatoprotective effects in rats by *M. oleifera* were reported by Fakurazi et al. [32]. Our results are in accordance with that published data. Furthermore, it is necessary to measure more inflammation and fibrosis markers and oxidative stress molecules to obtain a wider spectrum about the potential of *M. oleifera* in cells transfected with HBV.

The expression of *CAT* was decreased in transfected Huh7 cells with HBV (Figure 3(c)). After *M. oleifera* treatment, the *CAT* expression was reestablished. There are studies *in vivo* and *in vitro* about antioxidant components of *M. oleifera* leaves [33]. Sreelatha and Padma [34] reported an antioxidative activity in vitro arisen from phenol and flavonoid components of different parts of the *M. oleifera* plant. *In vitro*, the higher the total content of polyphenols the higher the antioxidant activity [35, 36]. Verma et al. showed potential of *M. oleifera* to increase enzymatic levels of catalase and superoxide dismutase in rats [37]. It is known that infection with HBV decreases antioxidative components [38] and increases reactive oxygen species (ROS) and prooxidants [39]. The endoplasmic reticulum of the host cell is saturated because of the high amount of proteins being synthesized for the viral particle assembly. This provokes an imbalance in the transport of calcium to the mitochondria, which results in inflammation, oxidative stress, and increase of ROS [39–42]. *M. oleifera* acts on the side of antioxidants restoring the imbalance by increasing *CAT* expression. Furthermore, it would be interesting to study the protein expression of catalase and the expression of other anti- and prooxidative components, which could be modified in response to *M oleifera*.

The gene expression of *IFNβ1* was increased in Huh7 cells transfected with both genotypes of HBV and further decreased with increasing *M. oleifera* treatment (Figure 3(d)). It is reported that cells in response to a virus attack enhance IFN*β* production as a defense [43, 44]. Patients infected with HBV genotype C are lower responders for IFN*α* treatment, probably the same resistance is for IFN*β* treatment. In this study, in spite that *M. oleifera* treatment decreased mRNA-IFN*β*, the decrease in cellular pgRNA and in the HBV-DNA observed in the supernatant from Huh7-transfected cells with genotype C could be influenced by a different mechanism not by IFN*β* production. In the case of genotype H, there are no reports about the reaction to interferon type 1 treatment. In this regard, we have previous unpublished results showing that genotype H responds to IFN*β* in a similar way as genotype A which is more sensitive to IFN*α* treatment. Probably, the decrease observed in the mRNA-IFN*β* nullifies the antiviral activity of *M. oleifera* in Huh7 cells transfected with genotype H.

Also, in this study, *M. oleifera* showed a tendency to decrease IL-6 in Huh7 cells transfected with genotypes C and H (Figure 4). This interleukin is produced mainly by Kupffer cells and regulates HBV gene expression and replication shortly after infection [45]. Complete neutralization of IL-6 for treatment of certain diseases may represent a risk if the patient is HBV infected [45]. Nevertheless, a balance of IL-6 is very important since transcriptionally enhanced IL-6 strongly facilitates the activation of human hepatic stellate cells favoring the advance of liver fibrosis. In animal models using rats with hepatotoxicity and diabetes induced by streptozotocin, *M. oleifera* showed a hepatoprotective, anti-inflammatory, and lipid-lowering effect in the treated diabetic rats compared to controls. IL-6 was returned to normal levels as well as TNF-*α* in the rats treated with Moringa [46]. It is possible that in patients with chronic hepatitis B, supplementation with Moringa could delay fibrosis progression.

## 5. Conclusion

Aqueous leaf extract of *M. oleifera* had no cytotoxic effect on Huh7 cells at selected concentrations (30, 45, and 60 *μ*g/mL) but modulated the pgRNA levels and HBsAg secretion positively in Huh7 cells transfected with genotypes C and H of HBV. The antiviral effect determined by reduction of DNA-HBV in the supernatant was only evidenced by genotype C and probably was independent of *IFNβ1* activity. In spite of the differences in the antiviral activity between genotypes C and H, gene expression modification of *CTGF*, *TGF-β1*, and *CAT* was modulated by *M. oleifera* treatment, favoring an antifibrotic, antioxidative, and anti-IL-6 environment. Further experiments are necessary to extend the obtained benefits of *M. oleifera* on HBV.
